# Supplementary material for: Design of crystal-like aperiodic solids with selective disorder–phonon coupling
Source: Nat Commun. 2016 Feb 4;7:10445. doi: 10.1038/ncomms10445 (PMC4742854; doi:10.1038/ncomms10445)
Supplement: Supplementary Information — Supplementary Figures 1-42, Supplementary Tables 1-5, Supplementary Notes 1-3, Supplementary Methods and Supplementary References [file ncomms10445-s1.pdf]

Supplementary Figures

S_1 and S_3

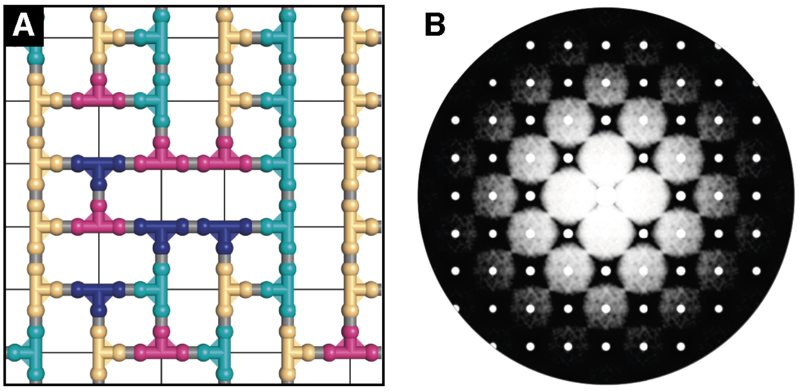

Procrystalline symmetry:

Pauling number (p)	1.00
Point group of parent node	D_{4h}
Point group of node	C_{2v}

Supplementary Figure 1: (A) Configuration and (B) corresponding (hk)* diffraction pattern for the **S_3** procrystalline system.

Known examples for $\boldsymbol{S_1}$ and $\boldsymbol{S_3}$:

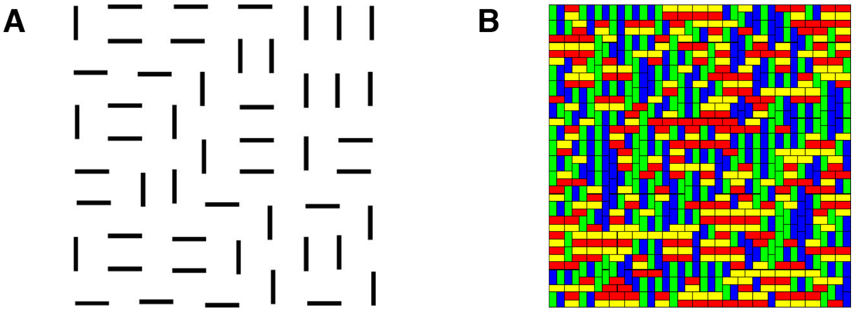

Supplementary Figure 2: (A) Anderson's resonating valence bond (RVB) description of electronic states in cuprate superconductors. Adapted from Ref. 1. (B) A tiling of dominos – a 1×2 rectangle – on a square. Adapted from Ref. 2.

S_2C

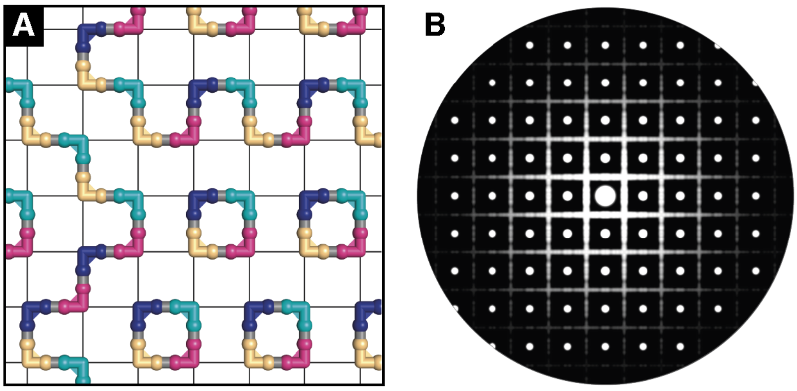

Procrystalline symmetry:

Pauling number (p)	1.00
Point group of parent node	D_{4h}
Point group of node	C_{2v}

Supplementary Figure 3: (A) Configuration and (B) corresponding (hk)* diffraction pattern for the $\mathbf{S_2C}$ procrystalline system.

Known examples for $\boldsymbol{S_2C}$:

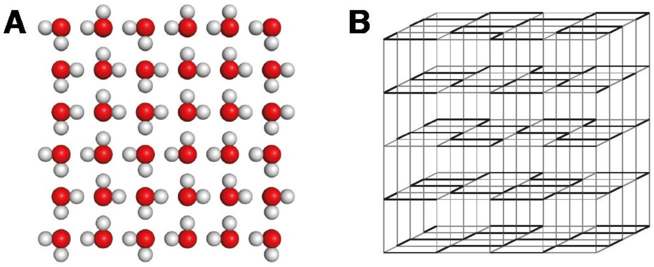

Supplementary Figure 4: (A) A square ice configuration with each molecule accepting two hydrogen bonds and donating two hydrogen bonds. Adapted from Ref. 3. (B) Anion order observed in perovskite oxynitrides –$SrNbO_2N$ – with cis oriented oxygens and nitrogen atoms around square planar niobium nodes. Adapted from Ref. 4. Reprinted by permission from Macmillan Publishers Ltd: *Nature Chemistry* (Yang, M. *et al.* Anion order in perovskite oxynitrides. **3**, 47–52), copyright (2011).

$\mathbf{S_2T}$

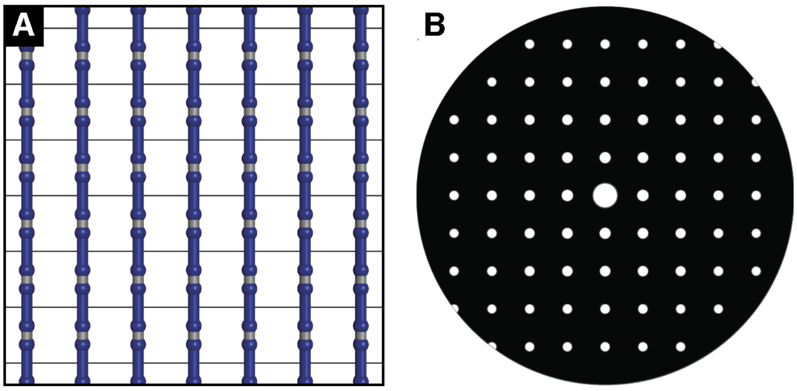

Procrystalline symmetry:

Pauling number (p)	0.50
Point group of parent node	D_{4h}
Point group of node	D_{2h}

Supplementary Figure 5: (A) Configuration and (B) corresponding (hk)* diffraction pattern for the $\mathbf{S_2T}$ procrystalline system. The lattice ordered and has rectangular symmetry. In the absence of lattice relaxation, this symmetry lowering is evident only in the distribution of Bragg intensities (not discernible here).

H_1 and H_2

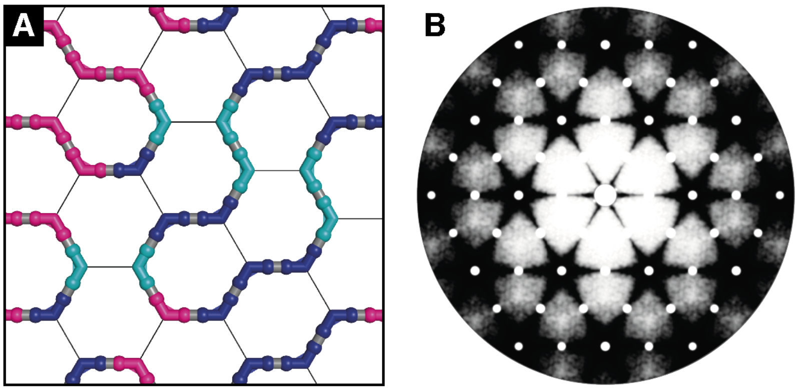

Procrystalline symmetry:

Pauling number (p)	1.06
Point group of parent node	D_{3h}
Point group of node	C_{2v}

Supplementary Figure 6: (A) Configuration and (B) corresponding (hk)* diffraction pattern for the $\mathbf{H_2}$ procrystalline system.

Known examples for $\boldsymbol{H_1}$ and $\boldsymbol{H_2}$:

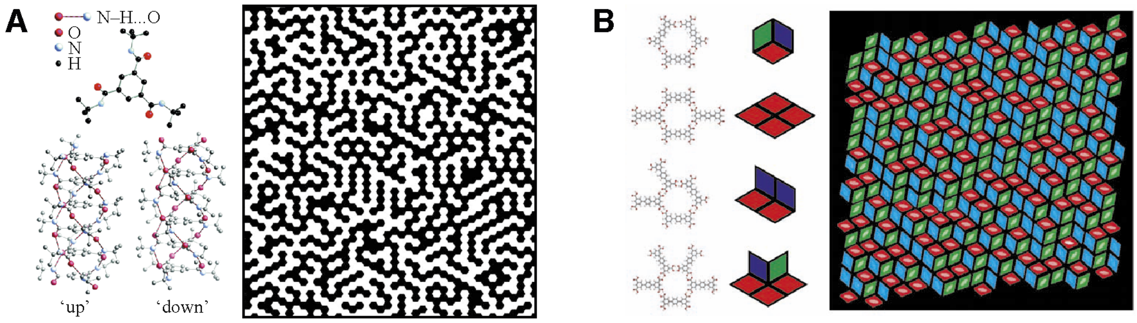

Supplementary Figure 7: (A) A representation of columnar polarisation states in solid-phase tris-*tert*-butyl-1,3,5-benzene tricarboxamide, giving a physical realisation of the Ising triangular anti-ferromagnet. Adapted from Ref. 5. Reproduced with permission of the International Union of Crystallography. (B) A hydrogen-bonded network of *p*-terphenyl-3,4,3',5'-tetracarboxylicacid molecules on pyrolytic graphite – mapping onto rhombus tiling. Adapted from Ref 6. From Blunt, M. O. *et al.* Random tiling and topological defects in a two-dimensional molecular network. *Science* **322**, 1077–1081 (2008). Reprinted with permission from AAAS.

T_1 and T_5

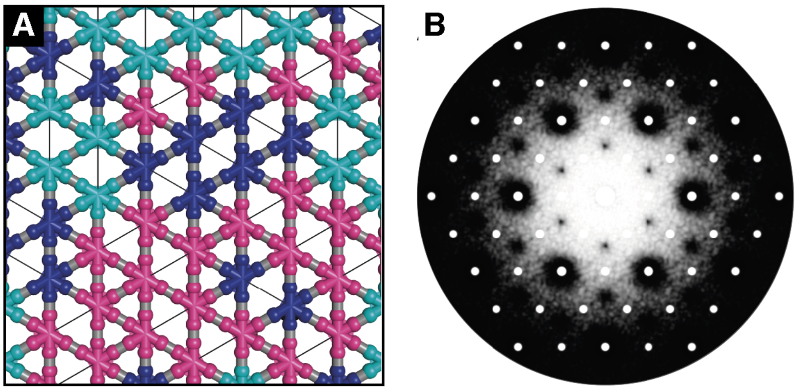

Procrystalline symmetry:

Pauling number (p)	0.75
Point group of parent node	D_{6h}
Point group of node	C_{2v}

Supplementary Figure 8: (A) Configuration and (B) corresponding (hk)* diffraction pattern for the $\mathbf{T_5}$ procrystalline system.

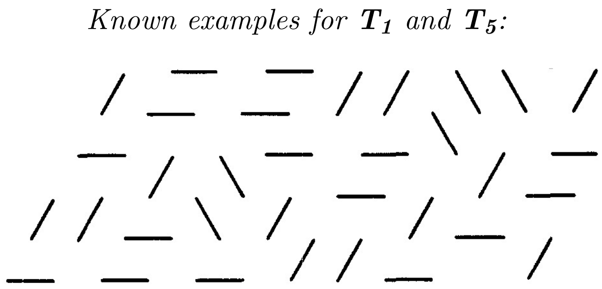

Supplementary Figure 9: Anderson's resonating valence bond (RVB) description of singlet formation for a d^1 triangular lattice; his specific example was TaS_2 Adapted from Ref. 7. Reprinted from Materials Research Bulletin, **8**, P. W. Anderson, Resonating valence bonds: A new kind of insulator?, 153–160, copyright (1973) with permission from Elsevier.

T_2O and T_4O

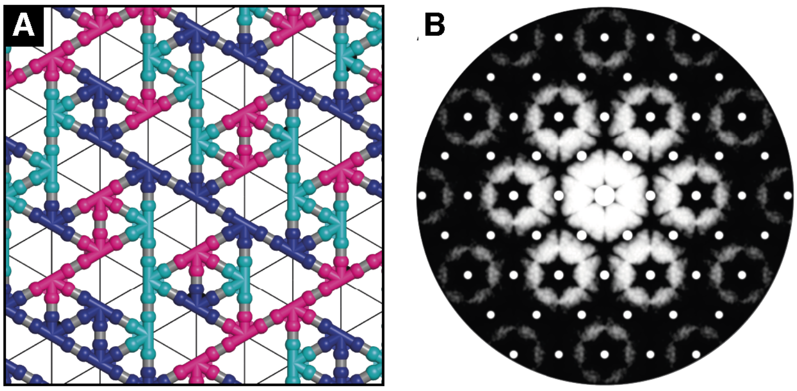

Procrystalline symmetry:

Pauling number (p)	0.75
Point group of parent node	D_{6h}
Point group of node	C_{2v}

Supplementary Figure 10: (A) Configuration and (B) corresponding (hk)* diffraction pattern for the **T_4O** procrystalline system.

T_2M and T_4M

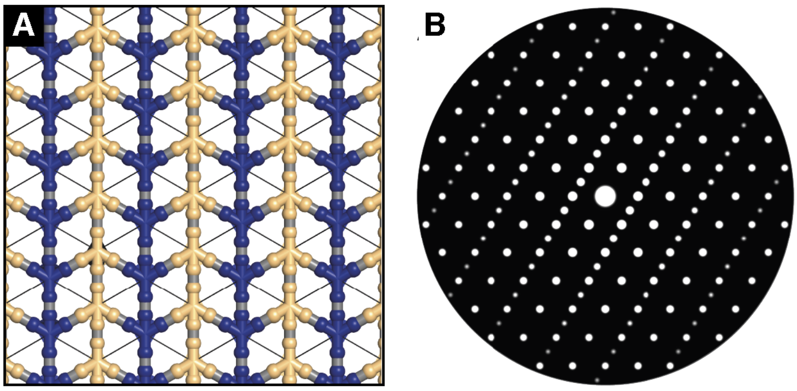

Procrystalline symmetry:

Pauling number (p)	0.75
Point group of parent node	D_{6h}
Point group of node	C_{2v}

Supplementary Figure 11: (A) Configuration and (B) corresponding (hk)* diffraction pattern for the $\mathbf{T_4M}$ procrystalline system. This lattice is ordered, with rectangular symmetry.

T_2P and T_4P

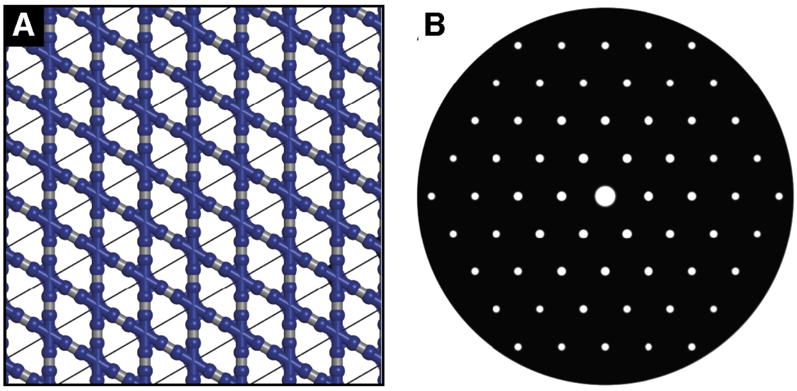

Procrystalline symmetry:

Pauling number (p)	0.38
Point group of parent node	D_{6h}
Point group of node	D_{2h}

Supplementary Figure 12: (A) Configuration and (B) corresponding (hk)* diffraction pattern for the $\mathbf{T_4P}$ procrystalline system. This lattice is ordered, with rectangular symmetry.

Known example for $\boldsymbol{T_2P}$ and $\boldsymbol{T_4P}$:

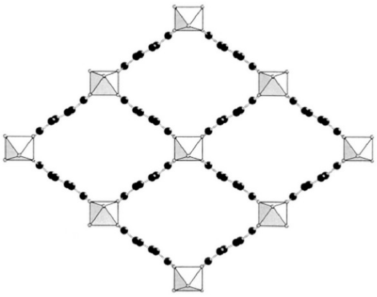

Supplementary Figure 13: MIL-53 – A Metal-Organic Framework (MOF) with the same topolgy as the $\mathbf{T_4P}$ procrystalline phase. Adapted from Ref. 8. Copyright 2004 Wiley-VCH Verlag GmbH & Co. KGaA, Weinheim.

$\mathbf{T_3V}$

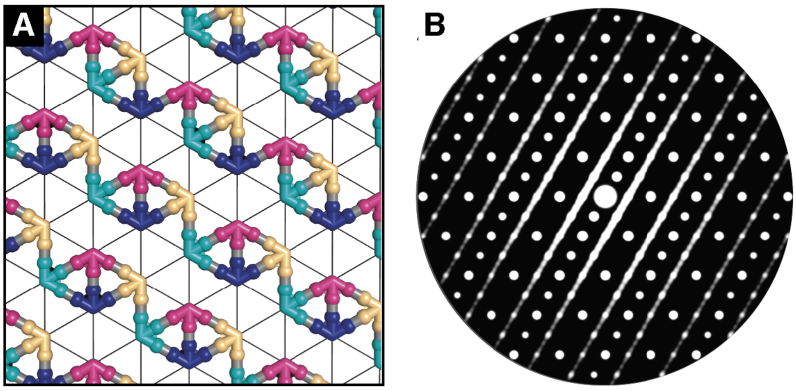

Procrystalline symmetry:

Pauling number (p)	0.75
Point group of parent node	D_{6h}
Point group of node	C_{2v}

Supplementary Figure 14: (A) Configuration and (B) corresponding $(hk)^*$ diffraction pattern for the $\mathbf{T_3V}$ procrystalline system. Though not long-range ordered, this lattice has rectangular symmetry at the macroscopic scale.

$\mathbf{T_3U_R}$

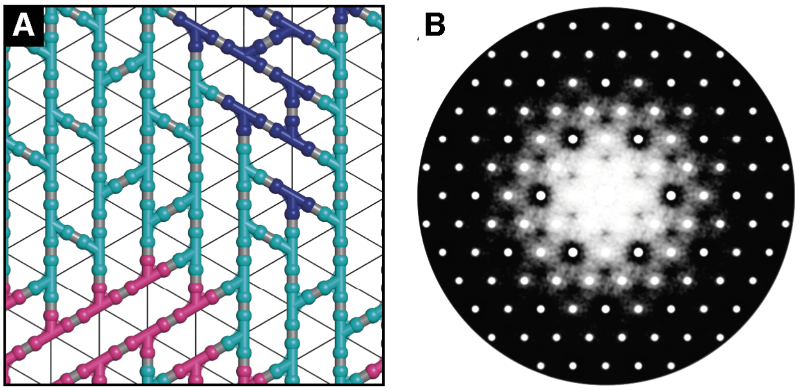

Procrystalline symmetry:

Pauling number (p)	1.50
Point group of parent node	D_{6h}
Point group of node	C_s

Supplementary Figure 15: (A) Configuration and (B) corresponding (hk)* diffraction pattern for the $\mathbf{T_3U_R}$ procrystalline system.

T_3U_X

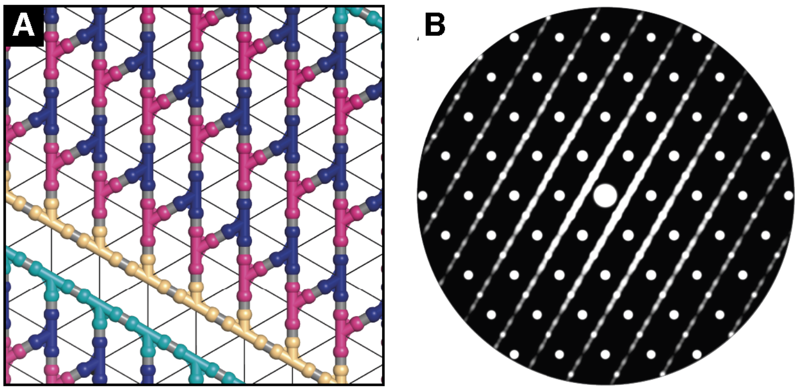

Procrystalline symmetry:

Pauling number (p)	0.75
Two dimensional point group of parent node	D_6
Two dimensional point group of node	C_1

Supplementary Figure 16: (A) Configuration and (B) corresponding $(hk)^*$ diffraction pattern for the $\mathbf{T_3U_X}$ procrystalline system. Though not long-range ordered, this lattice has rectangular symmetry at the macroscopic scale.

$\mathbf{T_3S}$

A configuration of $\mathbf{T_3S}$ is not realisable.

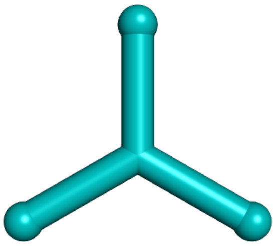

Procrystalline symmetry:

Pauling number (p)	0.25
Point group of parent node	D_{6h}
Point group of node	D_{3h}

Supplementary Figure 17: A description of the $\mathbf{T_3S}$ state, which can not form a configuration on the triangular lattice.

C_1 and C_5

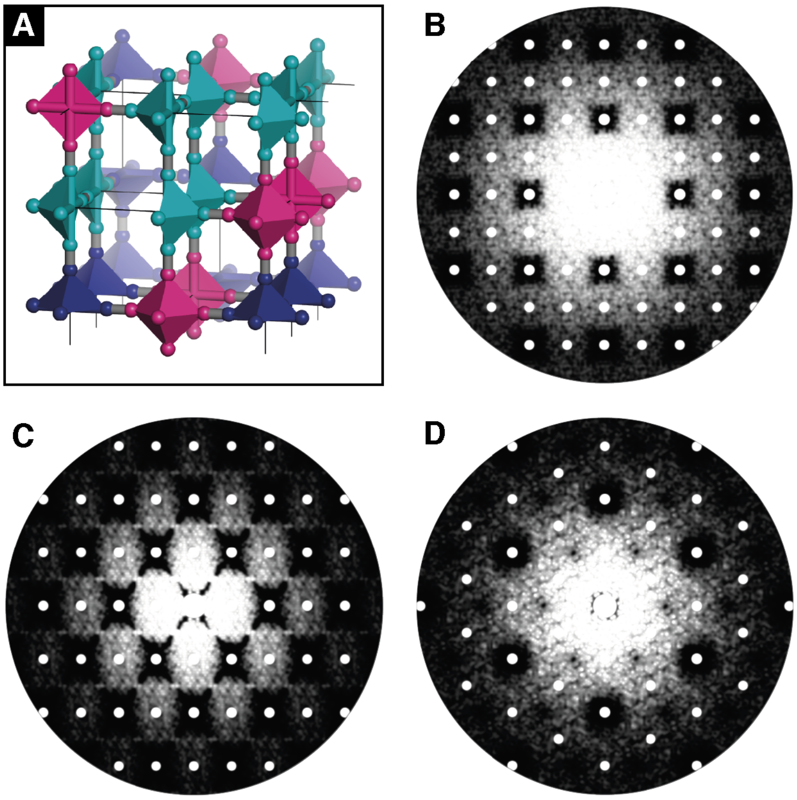

Procrystalline symmetry:

Pauling number (p)	0.75
Point group of parent node	O_h
Point group of node	C_{4v}

Supplementary Figure 18: (A) Configuration and corresponding diffraction patterns in the (B) (100)* (C) (110)* and (D) (111)* planes for the $\mathbf{C_5}$ procrystalline system.

C_2C and C_4C

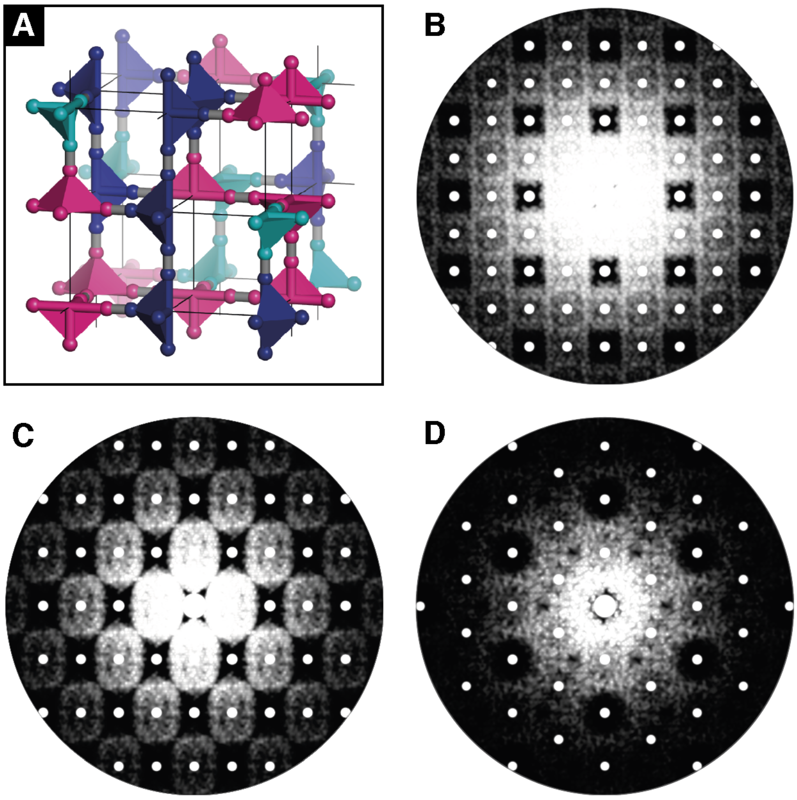

Procrystalline symmetry:

Pauling number (p)	1.50
Point group of parent node	O_h
Point group of node	C_{2v}

Supplementary Figure 19: (A) Configuration and corresponding diffraction patterns in the (B) (100)* (C) (110)* and (D) (111)* planes for the $\mathbf{C_4C}$ procrystalline system.

C_2T and C_4T

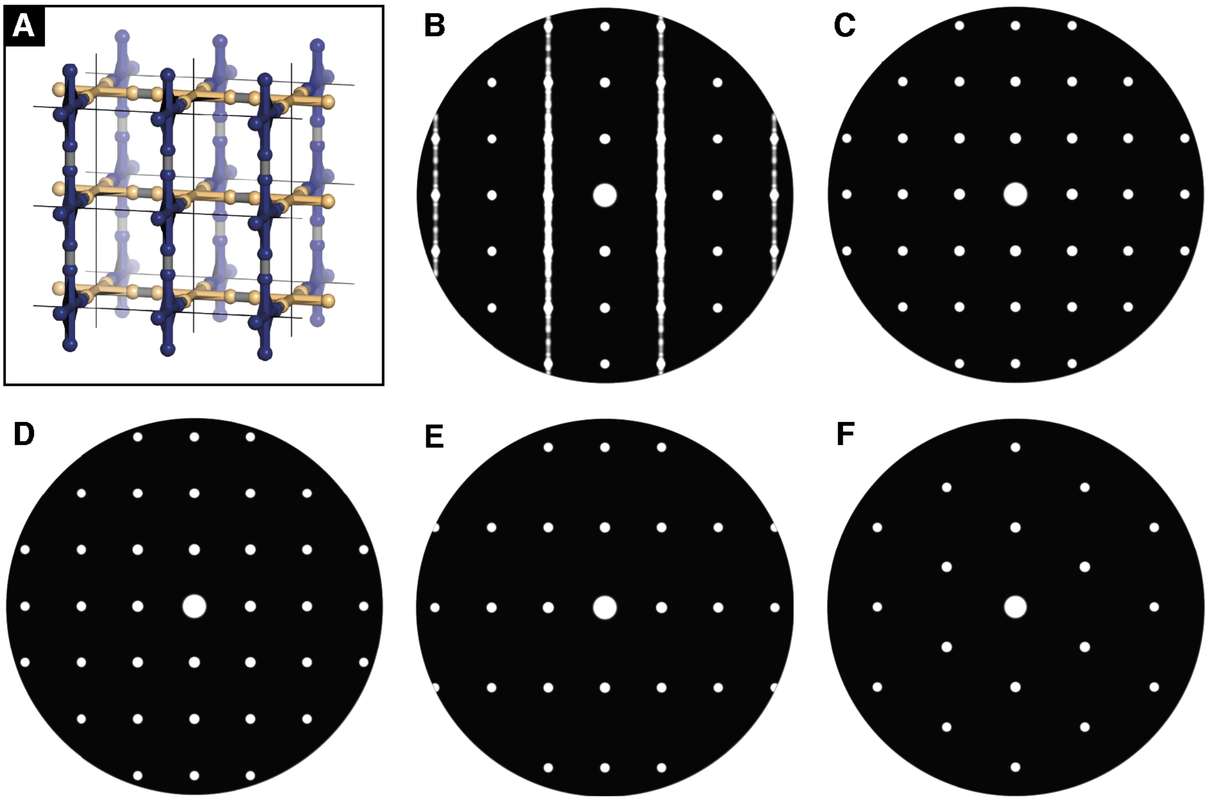

Procrystalline symmetry:

Pauling number (p)	0.38
Point group of parent node	O_h
Point group of node	D_{4h}

Supplementary Figure 20: (A) Configuration and corresponding diffraction patterns in the (B) (100)* (C) (010)* (D) (001)* (E) (110)* and (F) (111)* planes for the $\mathbf{C_4T}$ procrystalline system. Though not long-range ordered, this lattice has tetragonal symmetry at the macroscopic scale.

Known example for $\boldsymbol{C_2T}$ and $\boldsymbol{C_4T}$:

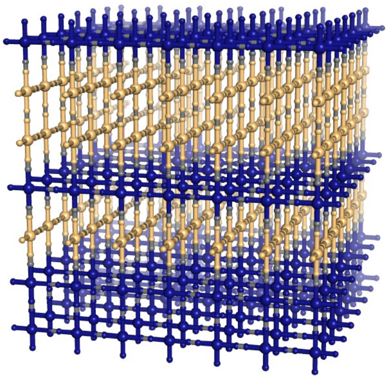

Supplementary Figure 21: The procrystalline model of $Pd(CN)_2/Pt(CN)_2$ as described in the main text.

C_3F

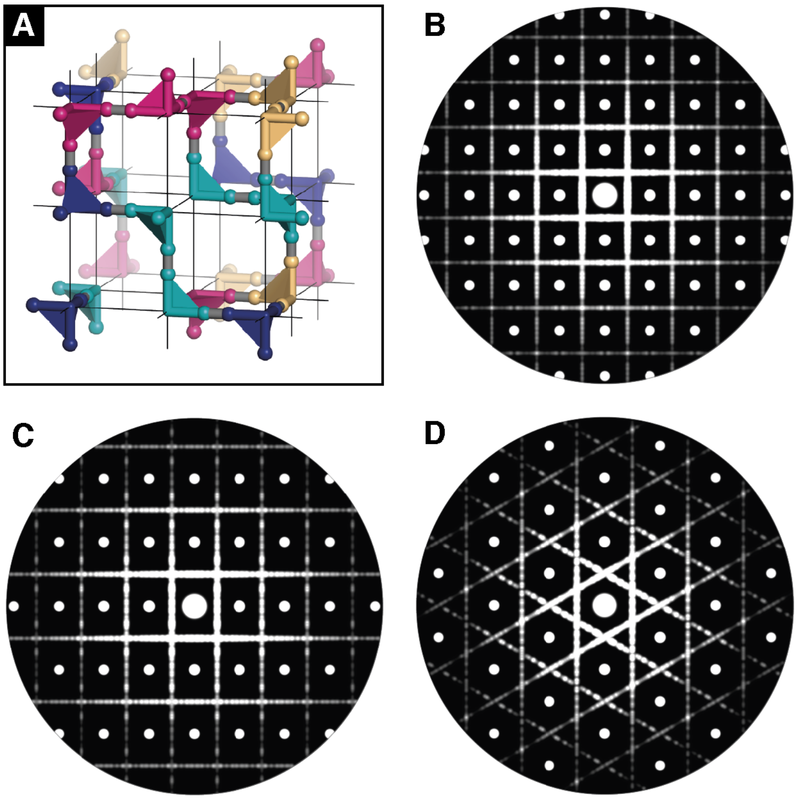

Procrystalline symmetry:

Pauling number (p)	1.00
Point group of parent node	O_h
Point group of node	C_{3v}

Supplementary Figure 22: (A) Configuration and corresponding diffraction patterns in the (B) (100)* (C) (110)* and (D) (111)* planes for the $\mathbf{C_3F}$ procrystalline system.

Known example for $\boldsymbol{C_3F}$:

A

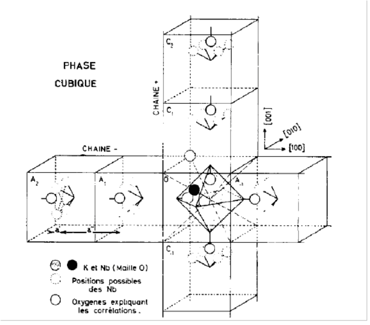

B

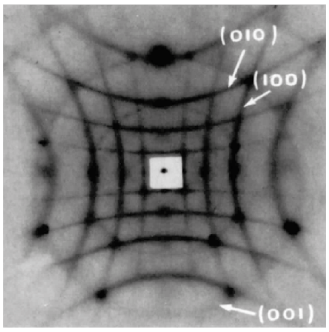

Supplementary Figure 23: (A) The structure of $KNbO_3$; isostructural to $BaTiO_3$. The procrystalline phase is realised through correlated displacements of the Nb atoms towards a face of the octahedral coordination environment – the $\langle 111 \rangle$ directions – such that no pair of neighbouring Nb atoms move toward each other in any of the $\langle 100 \rangle$ directions, as described in the main text. (B) The single crystal diffraction pattern of the cubic phase of $KNbO_3$ in the (100)* plane. This figure was distorted for use in Fig. 3(f) in the main text. Adapted from Ref. 9. Reproduced with permission of the International Union of Crystallography.

C_3M

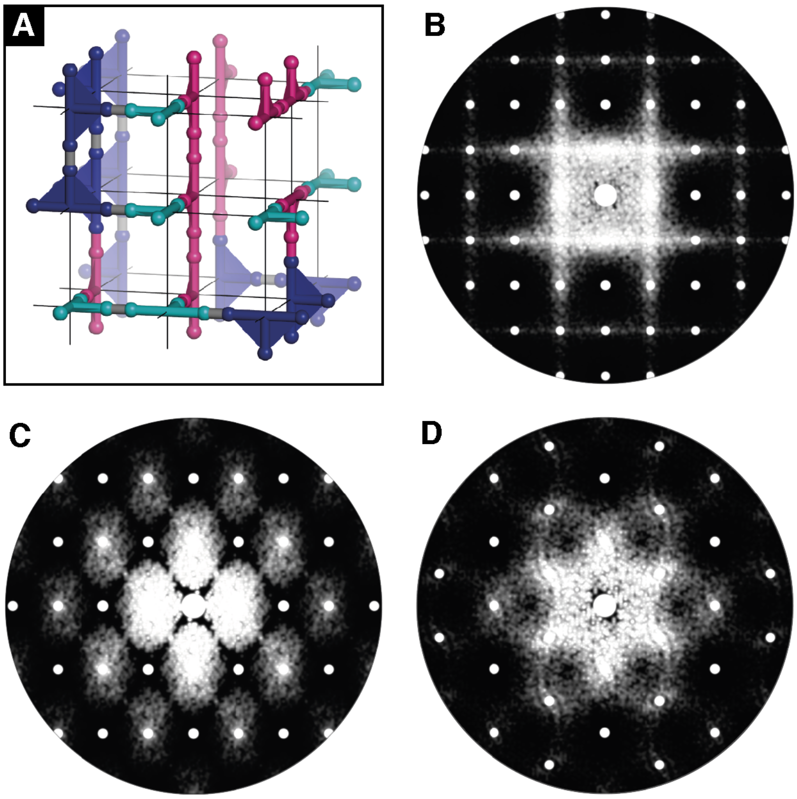

Procrystalline symmetry:

Pauling number (p)	1.50
Point group of parent node	O_h
Point group of node	C_{2v}

Supplementary Figure 24: (A) Configuration and corresponding diffraction patterns in the (B) (100)* (C) (110)* and (D) (111)* planes for the **C_3M** procrystalline system.

D_1 and D_3

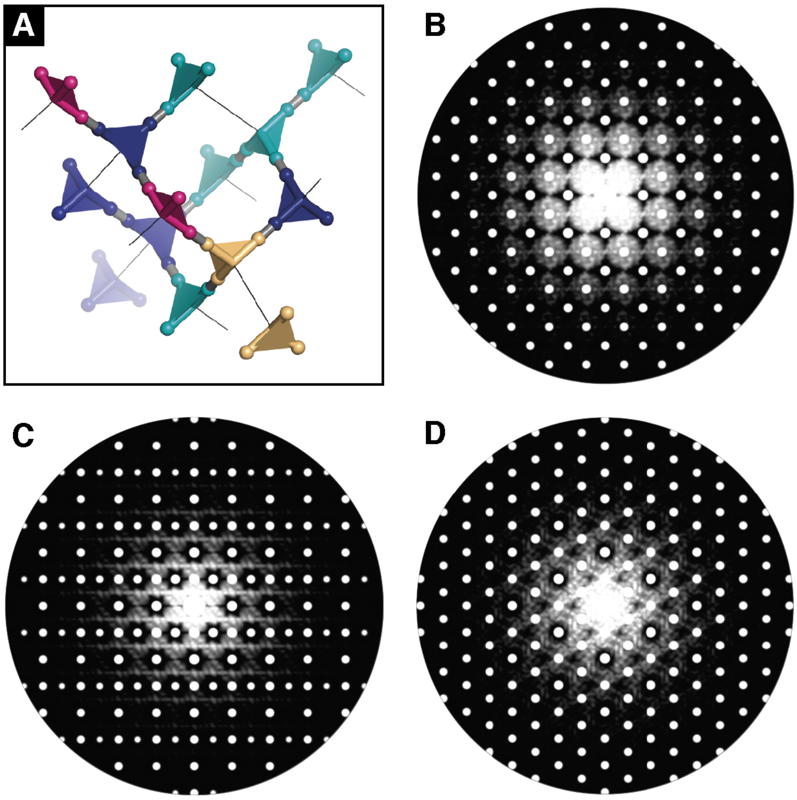

Procrystalline symmetry:

Pauling number (p)	1.00
Point group of parent node	T_d
Point group of node	C_{3v}

Supplementary Figure 25: (A) Configuration and corresponding diffraction patterns in the (B) (100)* (C) (110)* and (D) (111)* planes for two interpenetrating nets of the $\mathbf{D_3}$ procrystalline system.

$\mathbf{D_2}$

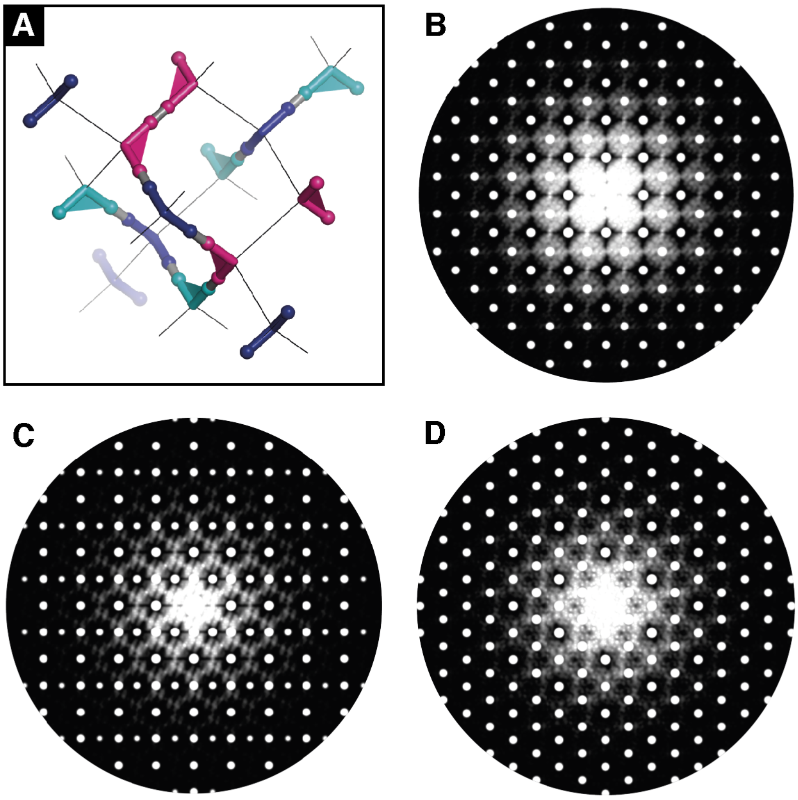

Procrystalline symmetry:

Pauling number (p)	1.50
Point group of parent node	T_d
Point group of node	C_{2v}

Supplementary Figure 26: (A) Configuration and corresponding diffraction patterns in the (B) (100)* (C) (110)* and (D) (111)* planes for for two interpenetrating nets of the $\mathbf{D_2}$ procrystalline system.

Known examples for $\boldsymbol{D_2}$*:*

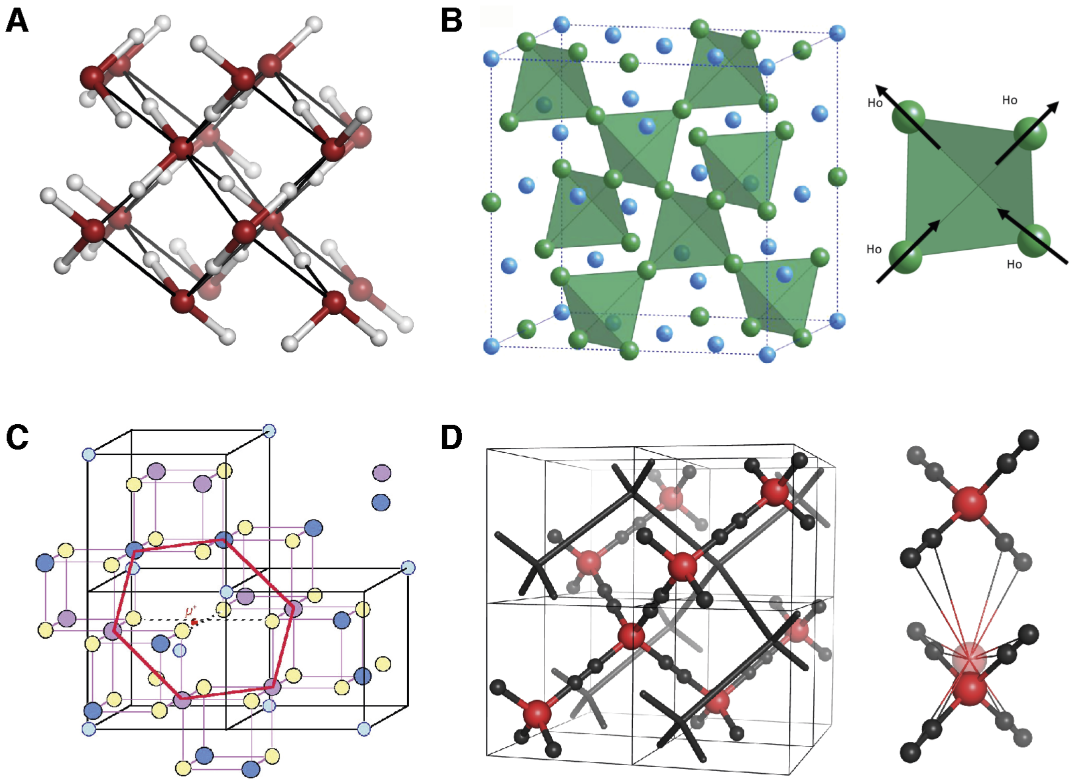

Supplementary Figure 27: (A) A configuration of ice whereby each oxygen atom accepts two hydrogen bonds and donates two hydrogen bonds. (B) Magnetic ordering in the pyrochlore spin ice Ho_2CrSbO_7. Adapted from Ref. 10. Note that in this case it is not only is the spin arrangement, but also the distribution of Cr and Sb atoms that is described by the $\mathbf{D_2}$ lattice. (C) A configuration of LiV_2O_4 in which each vanadium tetrahedron contains two V^{3+} ions and two V^{4+} ions. Adapted from Ref. 11. Copyright IOP Publishing. Re-produced with permission. All rights reserved. (D) Correlated off-centering of cadmium in $Cd(CN)_2$, driven by $\mathbf{D_2}$-type orientational disorder of cyanide ions. Adapted from Ref. 12. Reprinted figure with permission from [Fairbank, V. E., Thompson, A. L., Cooper, R. I. & Goodwin, A. L. *Phys. Rev. B* **86**, 104113 (2012).] Copyright (2012) by the American Physical Society.

$\mathbf{P_1}$and $\mathbf{P_5}$

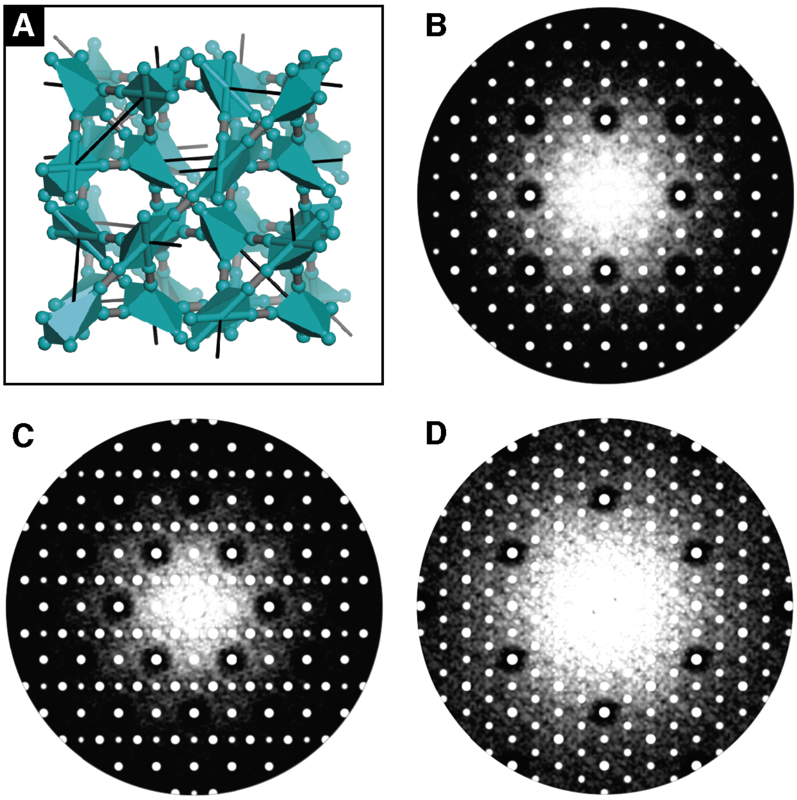

Procrystalline symmetry:

Pauling number (p)	0.75
Point group of parent node	D_{3d}
Point group of node	C_s

Supplementary Figure 28: (A) Configuration and corresponding diffraction patterns in the (B) (100)* (C) (110)* and (D) (111)* planes for two interpenetrating nets of the $\mathbf{P_5}$ procrystalline system.

Known example for $\boldsymbol{P_1}$ *and* $\boldsymbol{P_5}$*:*

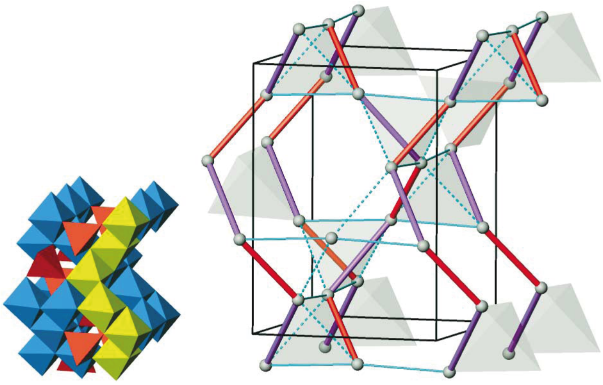

Supplementary Figure 29: Ti–Ti bond formation in the insulating phase of $MgTi_2O_4$ adopts an ordered configuration contained within the ensemble of configurations defined by $\mathbf{P_5}$. An RVB description of the metallic state at $T > 260$ K corresponds to the procrystalline model. Adapted from Ref. 13. Reprinted figure with permission from [Schmidt, M., Ratcliff II, W., Radaelli, P. G., Refson, K., Harrison, N. M. & Cheong, S. W. *Phys. Rev. Lett.* **92**, 056402 (2004).] Copyright (2004) by the American Physical Society

P_2C and P_4C

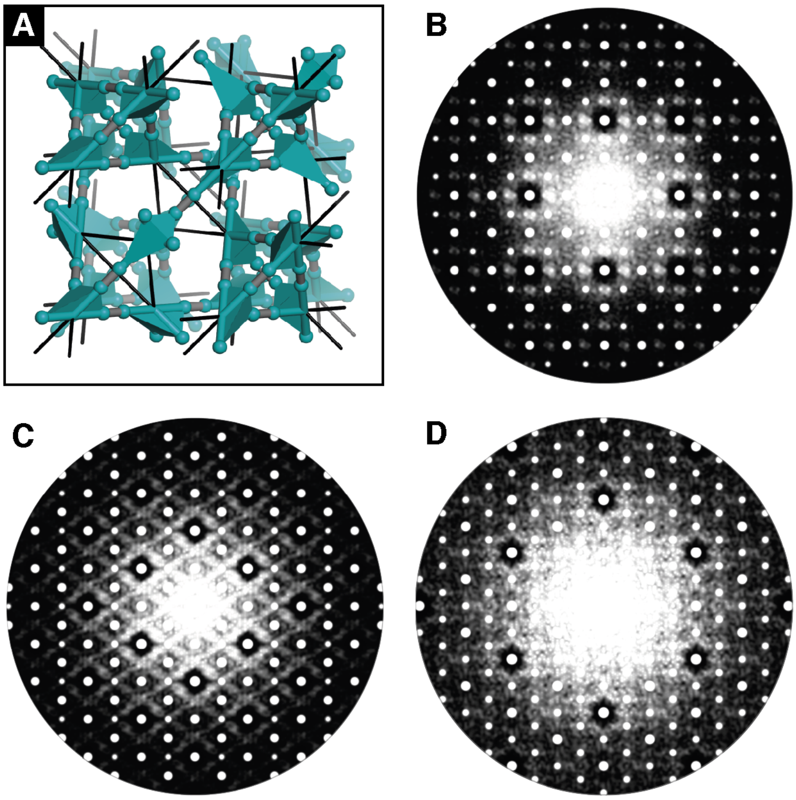

Procrystalline symmetry:

Pauling number (p)	0.75
Point group of parent node	D_{3d}
Point group of node	C_s

Supplementary Figure 30: (A) Configuration and corresponding diffraction patterns in the (B) (100)* (C) (110)* and (D) (111)* planes for two interpenetrating nets of the $\mathbf{P_4C}$ procrystalline system.

P_2O_R and P_4O_R

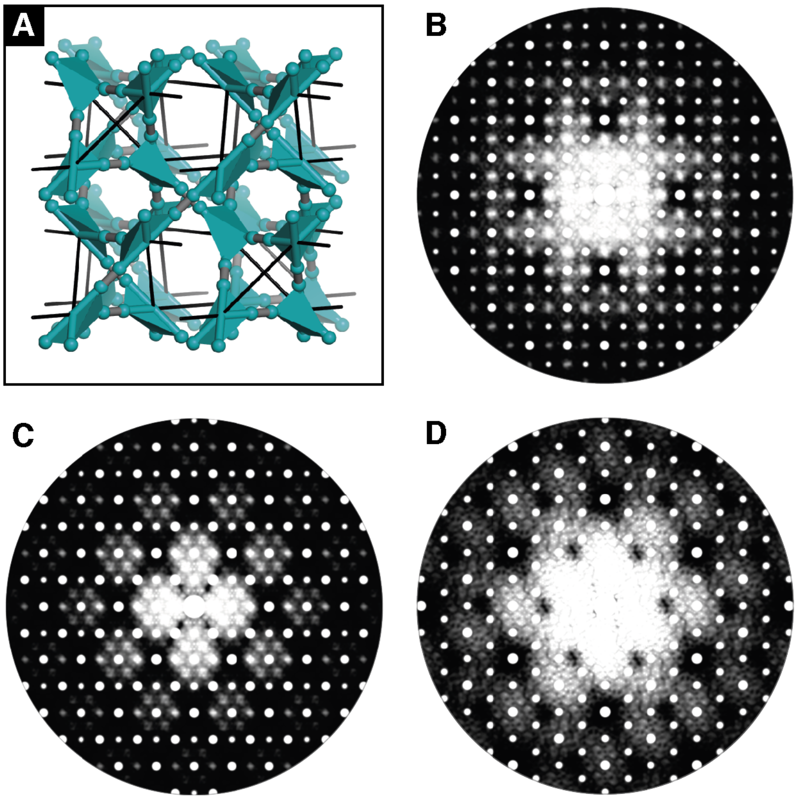

Procrystalline symmetry:

Pauling number (p)	0.75
Point group of parent node	D_{3d}
Point group of node	C_2

Supplementary Figure 31: (A) Configuration and corresponding diffraction patterns in the (B) (100)* (C) (110)* and (D) (111)* planes for two interpenetrating nets of the $\mathbf{P_4O_R}$ procrystalline system.

P_2O_X and P_4O_X

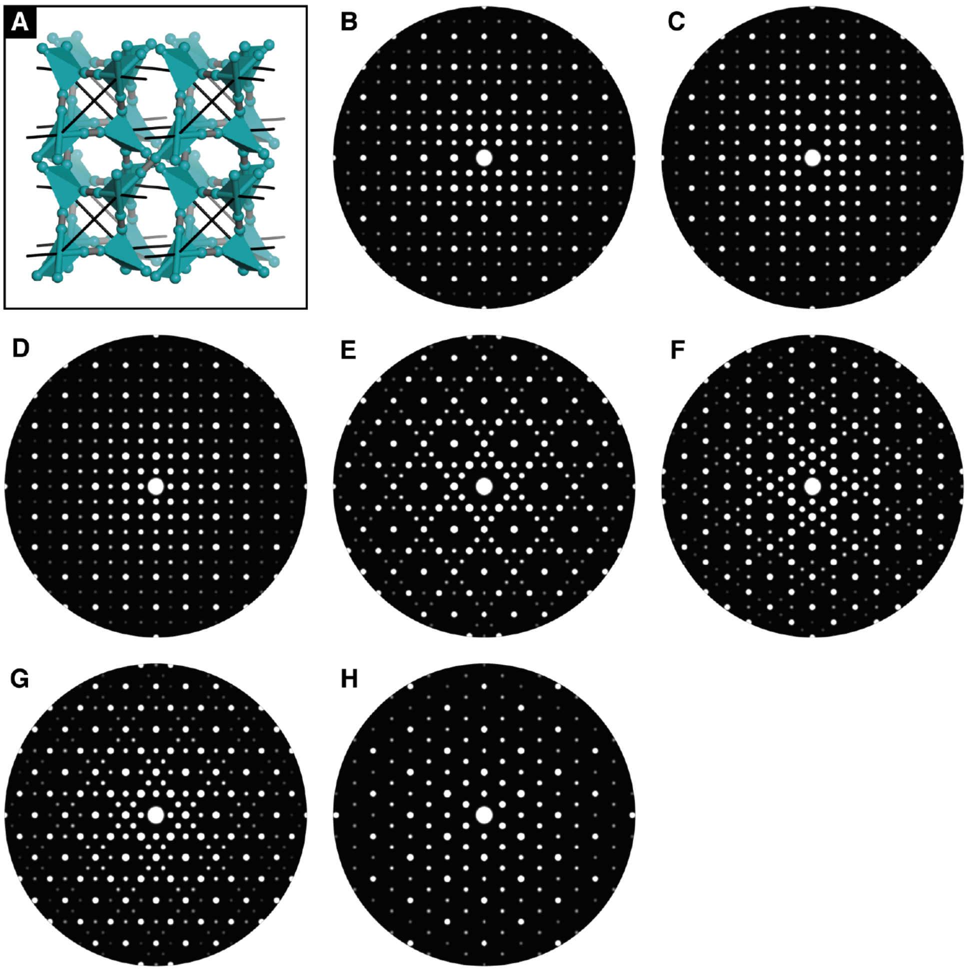

Procrystalline symmetry:

Pauling number (p)	0.38
Point group of parent node	D_{3d}
Point group of node	C_2

Supplementary Figure 32: (A) Configuration and corresponding diffraction patterns in the (B) (100)* (C) (010)* (D) (001)* (E) (110)* (F) (101)* (G) (011)* and (H) (111)* planes for two interpenetrating nets of the **P_4O_X** procrystalline system. This lattice is ordered, with tetragonal symmetry.

P_2T and P_4T

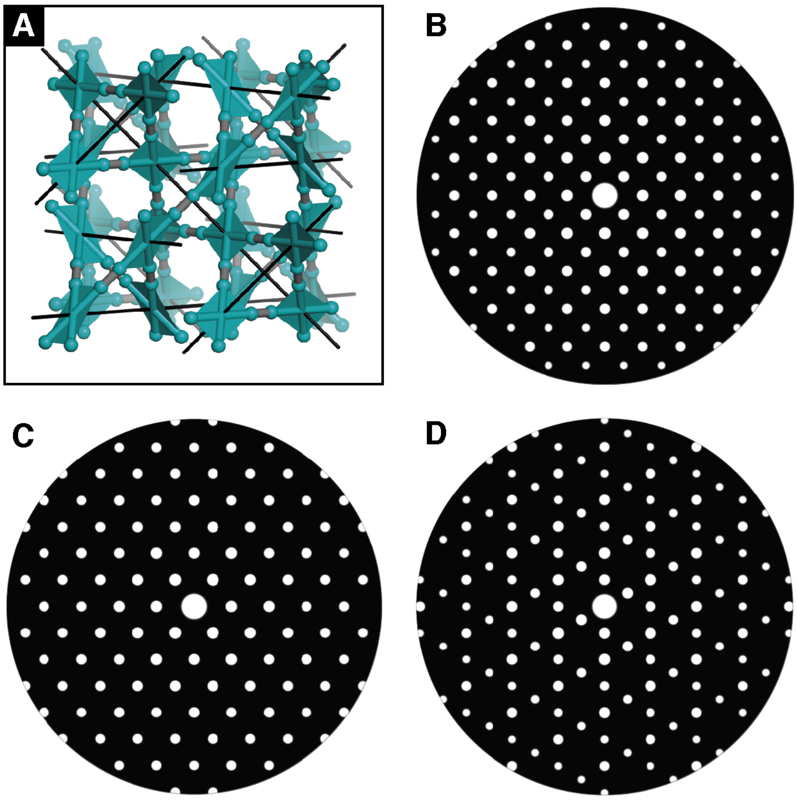

Procrystalline symmetry:

Pauling number (p)	0.38
Point group of parent node	D_{3d}
Point group of node	C_{2h}

Supplementary Figure 33: (A) Configuration and corresponding diffraction patterns in the (B) (100)* (C) (110)* and (D) (111)* planes for two interpenetrating nets of the $\mathbf{P_4T}$ procrystalline system. This configuration is ordered, with cubic symmetry

P_3A

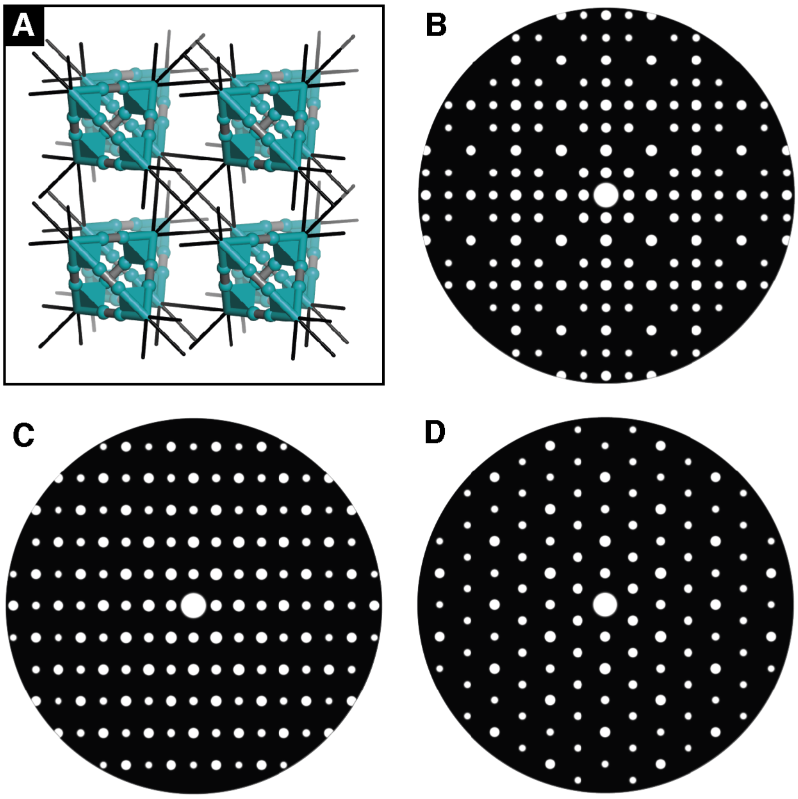

Procrystalline symmetry:

Pauling number (p)	0.25
Point group of parent node	D_{3d}
Point group of node	C_{3v}

Supplementary Figure 34: (A) Configuration and corresponding diffraction patterns in the (B) (100)* (C) (110)* and (D) (111)* planes for two interpenetrating nets of the $\mathbf{P_3A}$ procrystalline system. This lattice is ordered, with cubic symmetry.

P_3F

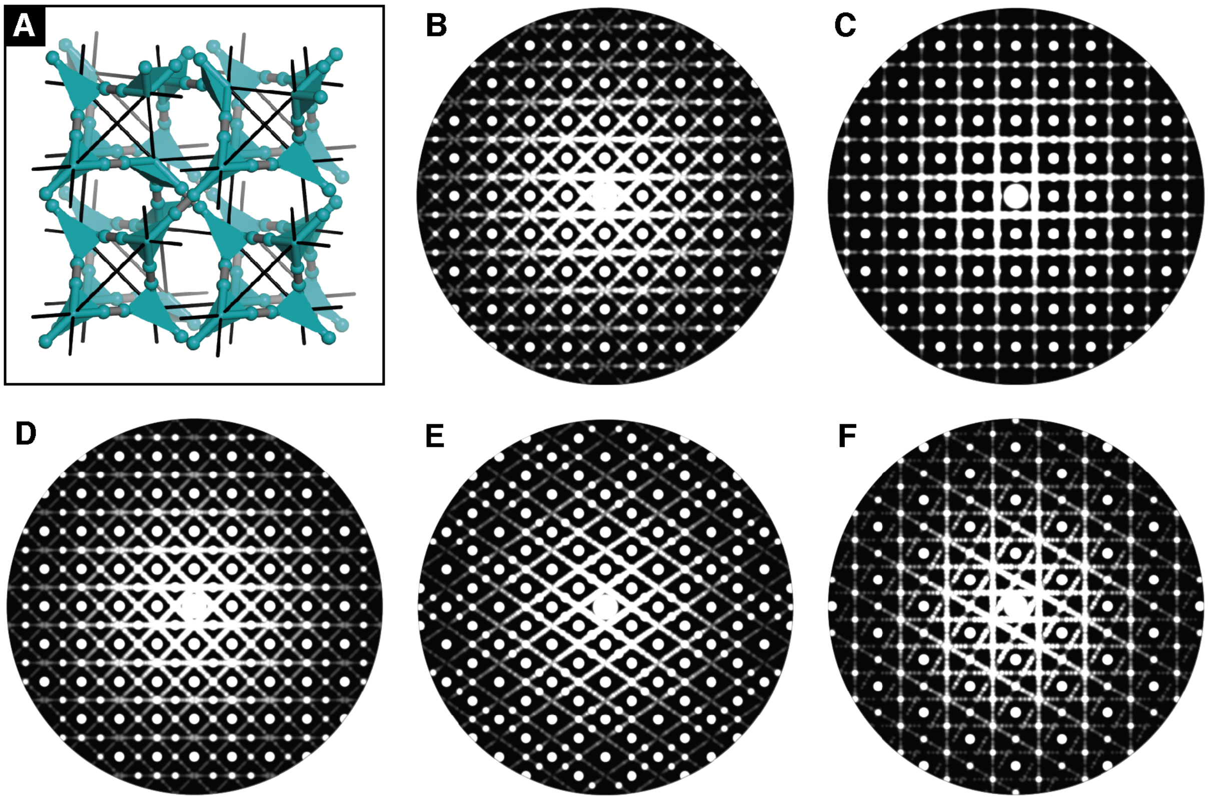

Procrystalline symmetry:

Pauling number (p)	0.75
Point group of parent node	D_{3d}
Point group of node	C_s

Supplementary Figure 35: (A) Configuration and corresponding diffraction patterns in the (B) (100)* (C) (010)* (D) (001)* (E) (110)* and (F) (111)* planes for two interpenetrating nets of the $\mathbf{P_3F}$ procrystalline system. Though not long-range ordered, this lattice has tetragonal symmetry at the macroscopic scale.

$\mathbf{P_3M_R}$

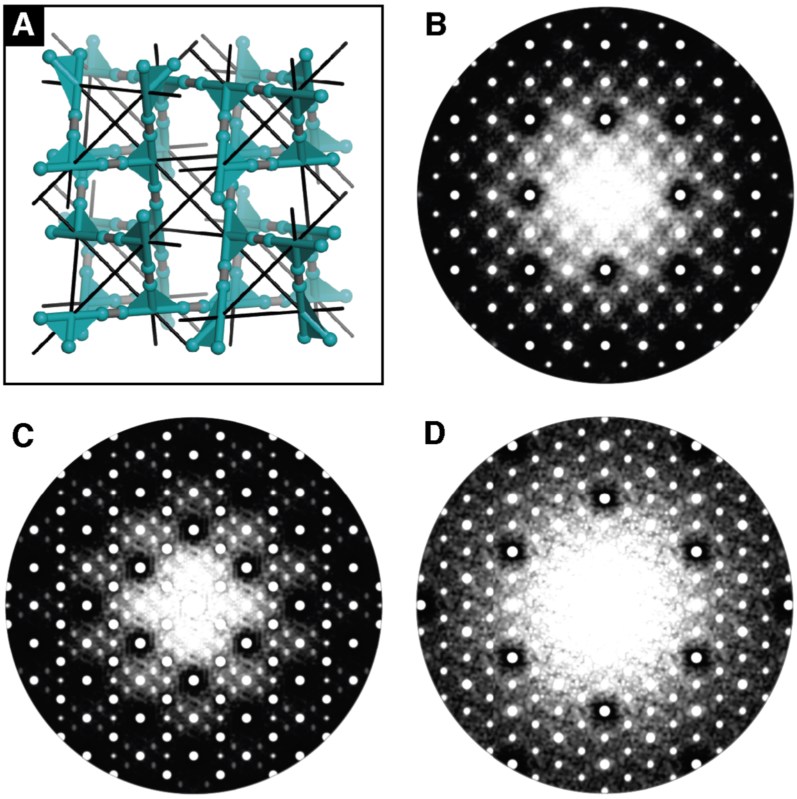

Procrystalline symmetry:

Pauling number (p)	1.50
Point group of parent node	D_{3d}
Point group of node	C_1

Supplementary Figure 36: (A) Configuration and corresponding diffraction patterns in the (B) (100)* (C) (110)* and (D) (111)* planes for two interpenetrating nets of the $\mathbf{P_3M_R}$ procrystalline system.

P_3M_X

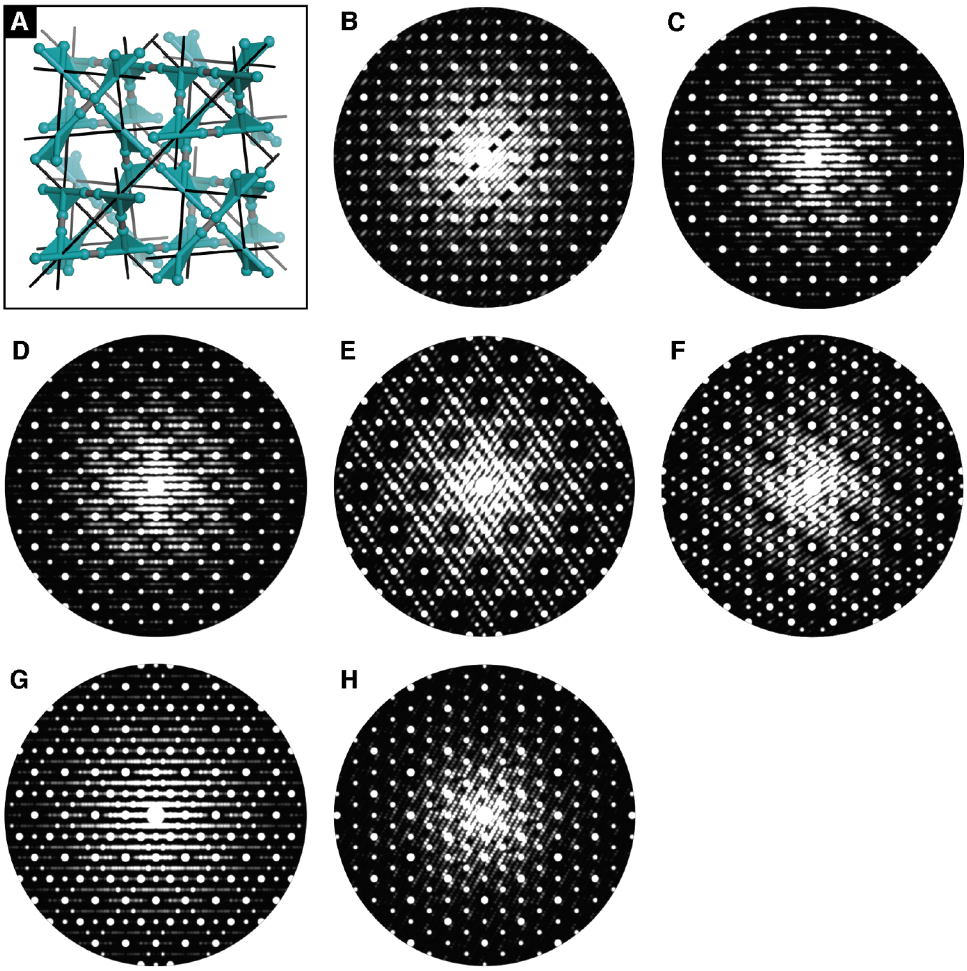

Procrystalline symmetry:

Pauling number (p)	0.75
Point group of parent node	D_{3d}
Point group of node	C_1

Supplementary Figure 37: (A) Configuration and corresponding diffraction patterns in the (B) (100)* (C) (010)* (D) (001)* (E) (110)* (F) (101)* (G) (011)* and (H) (111)* planes for two interpenetrating nets of the $\mathbf{P_3M_X}$ procrystalline system. Though not long-range ordered, this lattice has tetragonal symmetry at the macroscopic scale.

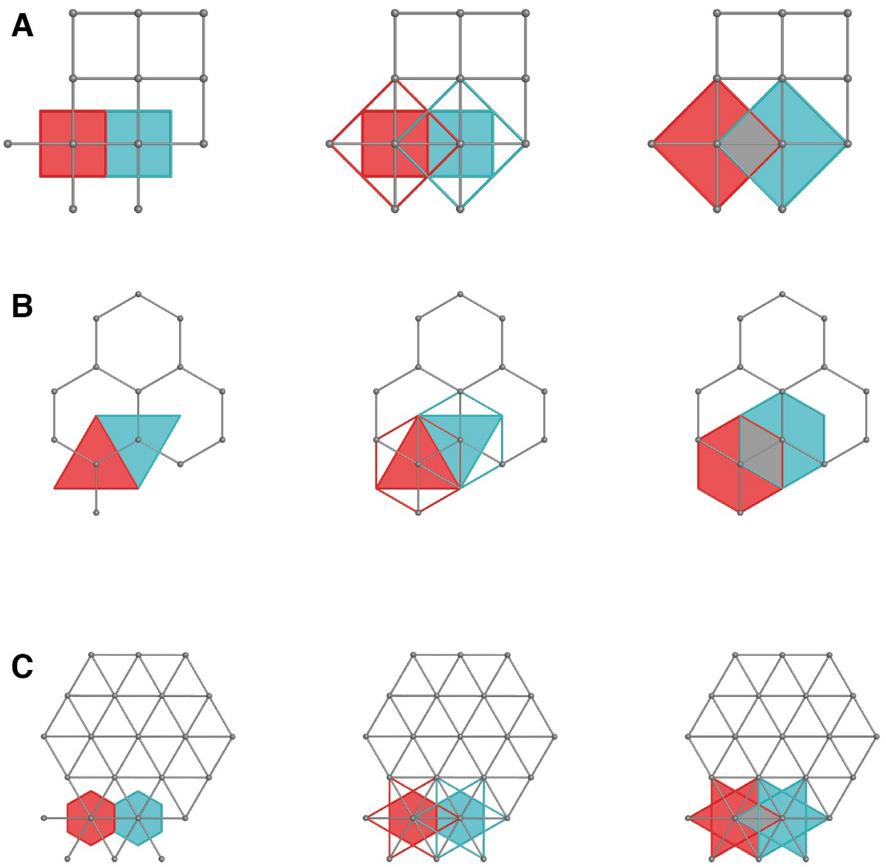

Supplementary Figure 38: Construction of neighbourhoods for 2D lattices: (A) square, (B) hexagonal, and (C) triangular. In each case a pair of adjacent Dirichlet-Voronoi cells are shown in the left-hand panel. The centre panel shows the augmentation of these cells to include correlated lattice points, and the right-hand panel shows the complete neighbourhoods. The overlapping region is coloured in grey in each case.

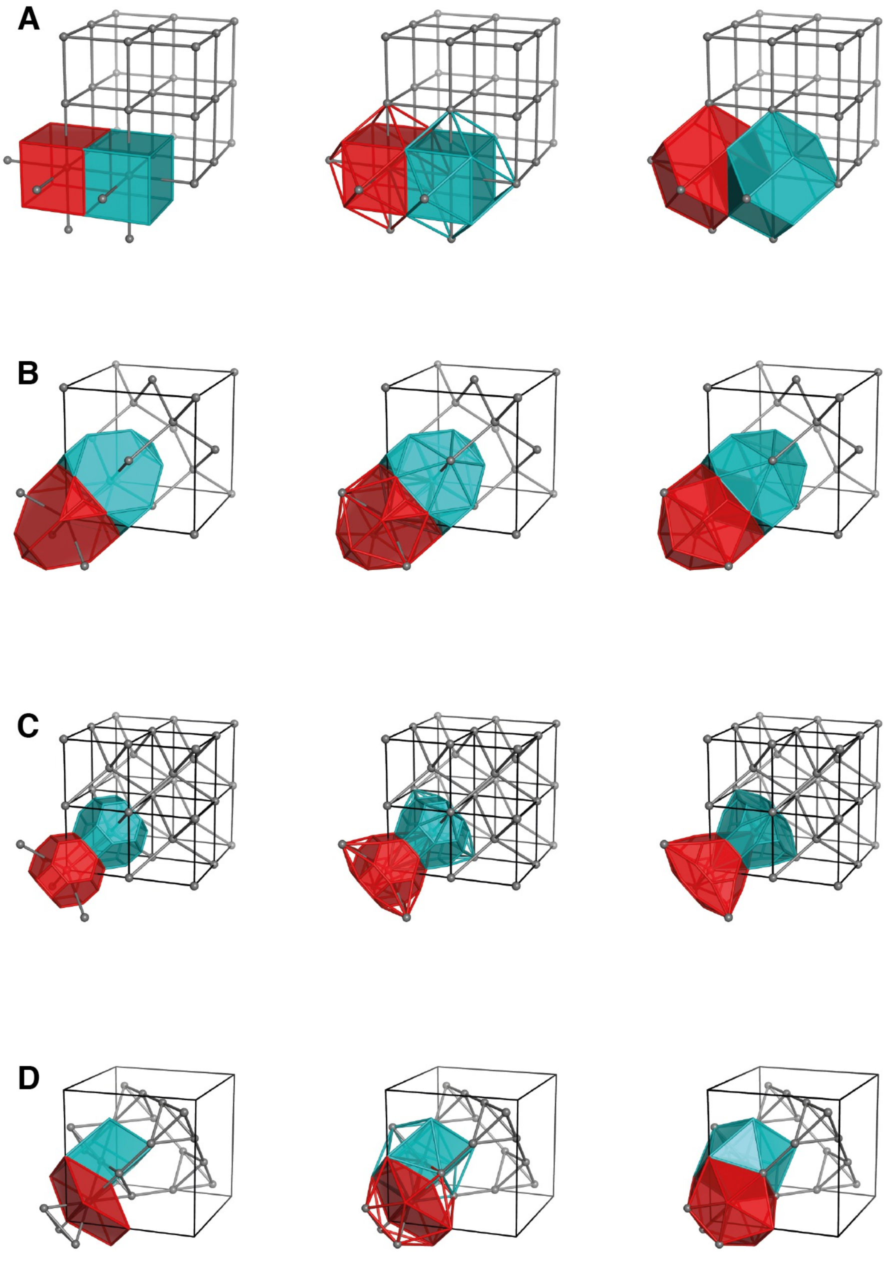

Supplementary Figure 39: Construction of neighbourhoods for 3D lattices: (A) cubic, (B) diamondoid, (C) doubly-interpenetrating diamondoid, (D) pyrochlore. The sequence of panels is as for Fig. 38.

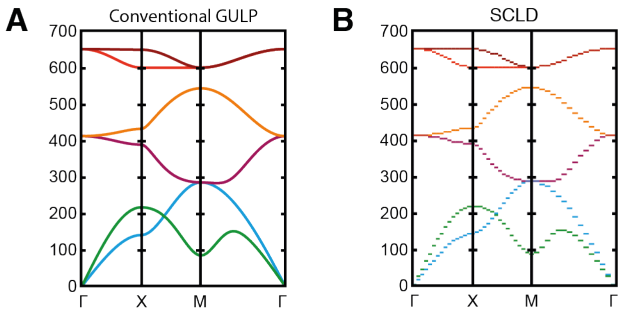

Supplementary Figure 40: (A) Phonon dispersion curves calculated for the mean-field case in the unit cell implementation of GULP. (B) Phonon dispersion curves calculated for the mean-field case in the supercell implementation of GULP (SCLD).

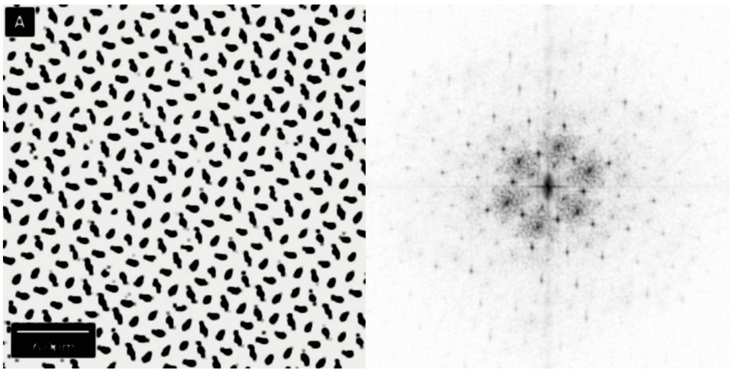

Supplementary Figure 41: (Left) STM image of molecules of p-terphenyl-3,5,3′,5′-tetracarboxylic acid (TPTC) assembled on pyrolytic graphite (Ref. 6), treated as described in the text, and (right) its Fourier transform.

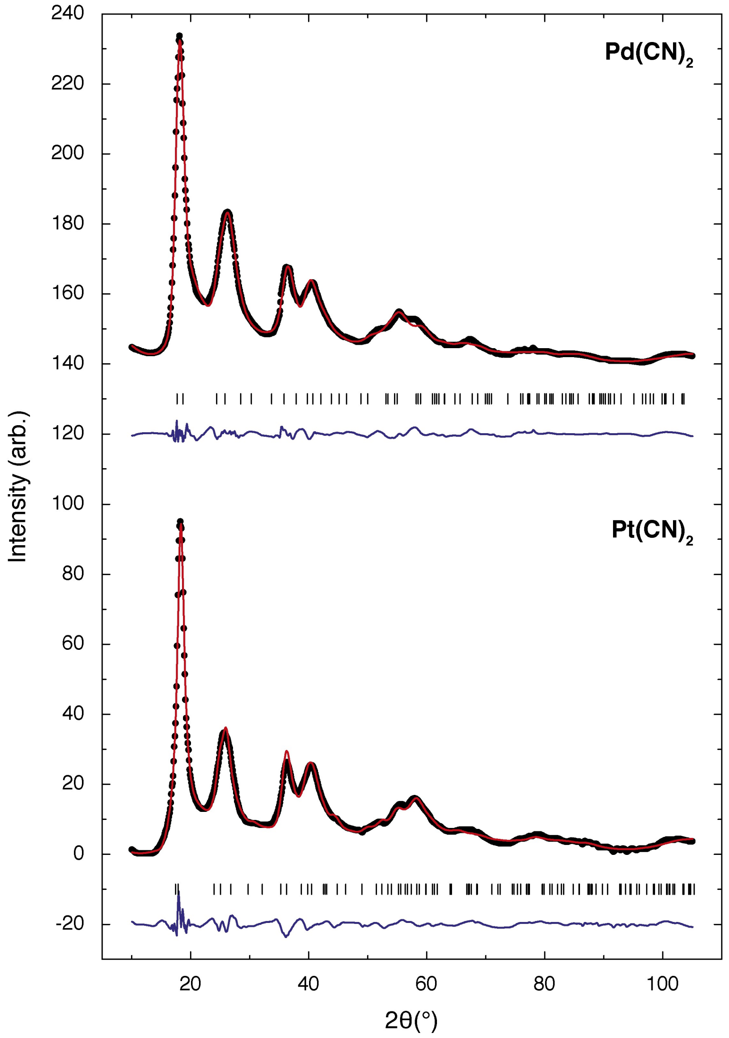

Supplementary Figure 42: Rietveld fits (red lines) to X-ray powder diffraction data from Ref. 14 (black points) for $Pd(CN)_2$ (top) and $Pt(CN)_2$ (bottom). Difference curve (data−fit) shown in blue; tick marks correspond to expected peak positions.

Supplementary Table 1: Details of supercell configurations used for Monte Carlo generation of procrystalline configurations. *When convergence of a procrystalline phase was not possible in a 40×40 supercell a 42×42 supercell was used. †A doubly interpenetrating network.

Lattice	Plane/space group	Lattice points/ unit cell	Supercell dimensions	Total number of lattice points
Square	$p4m$	1	40×40	1600
Hexagonal	$p3m1$	2	40×40	3200
Triangular	$p6m$	1	$40 \times 40^*$	1600
Cubic	$Pm\bar{3}m$	1	$20 \times 20 \times 20$	8000
Diamondoid†	$Pn\bar{3}m$	2	$10 \times 10 \times 10$	2000
Pyrochlore†	$Pn\bar{3}m$	4	$10 \times 10 \times 10$	4000

Supplementary Table 2: Notation used for identification of procrystalline configurations.

Lattice	Notation	Stereochemistry	Notation	Relevant Lattice(s)	Stereochemistry	Notation	Relevant Lattice(s)
Square	**S**	Cis	**C**	**S**, **C**	Facial	**F**	**C**
Hexagonal	**H**	Trans	**T**	**S**, **C**, **P**	Meridional	**M**	**C**
Triangular	**T**	Ortho	**O**	**T**	Acute Cis	**C**	**P**
Primitive Cubic	**C**	Meta	**M**	**T**	Obtuse Cis – Racemate	$\mathbf{O_R}$	**P**
Diamond	**D**	Para	**P**	**T**	Obtuse Cis – Enantiopure	$\mathbf{O_X}$	**P**
Pyrochlore	**P**	Vicinal	**V**	**T**	Acute Facial	**A**	**P**
		Unsymmetrical – Racemate	$\mathbf{U_R}$	**T**	Obtuse Facial	**F**	**P**
		Unsymmetrical – Enantiopure	$\mathbf{U_X}$	**T**	Meridional – Racemate	$\mathbf{M_R}$	**P**
		Symmetrical	**S**	**T**	Meridional – Enantiopure	$\mathbf{M_X}$	**P**

Supplementary Table 3: A summary of procrystalline phases including node symmetry and Pauling number. *For these chiral phases the absence of a point-symmetry element to interconvert enantiomeric nodes reduces the Pauling number by a factor of two.

Phase	Parent Symmetry	Node Symmetry	Pauling number (p)
$\mathbf{S_3}$	D_{4h}	C_{2v}	1.00
$\mathbf{S_2C}$	D_{4h}	C_{2v}	1.00
$\mathbf{S_2T}$	D_{4h}	D_{2h}	0.50
$\mathbf{H_2}$	D_{3h}	C_{2v}	1.06
$\mathbf{T_5}$	D_{6h}	C_{2v}	0.75
$\mathbf{T_4O}$	D_{6h}	C_{2v}	0.75
$\mathbf{T_4M}$	D_{6h}	C_{2v}	0.75
$\mathbf{T_4P}$	D_{6h}	D_{2h}	0.38
$\mathbf{T_3V}$	D_{6h}	C_{2v}	0.75
$\mathbf{T_3U_R}$	D_{6h}	C_s	1.50
$\mathbf{T_3U_X}$	D_{6h}	C_s*	0.75
$\mathbf{T_3S}$	D_{6h}	D_{3h}	0.25
$\mathbf{C_5}$	O_h	C_{4v}	0.75
$\mathbf{C_4C}$	O_h	C_{2v}	1.50
$\mathbf{C_4T}$	O_h	D_{4h}	0.38
$\mathbf{C_3F}$	O_h	C_{3v}	1.00
$\mathbf{C_3M}$	O_h	C_{2v}	1.50
$\mathbf{D_3}$	T_d	C_{3v}	1.00
$\mathbf{D_2}$	T_d	C_{2v}	1.50
$\mathbf{P_5}$	D_{3d}	C_s	0.75
$\mathbf{P_4C}$	D_{3d}	C_s	0.75
$\mathbf{P_4O_R}$	D_{3d}	C_2	0.75
$\mathbf{P_4O_X}$	D_{3d}	C_2*	0.38
$\mathbf{P_4T}$	D_{3d}	C_{2h}	0.38
$\mathbf{P_3A}$	D_{3d}	C_{3v}	0.25
$\mathbf{P_3F}$	D_{3d}	C_s	0.75
$\mathbf{P_3M_R}$	D_{3d}	C_1	1.50
$\mathbf{P_3M_X}$	D_{3d}	C_1*	0.75

Supplementary Table 4: Crystallographic details determined by powder X-ray diffraction for the crystalline approximant of $Pd(CN)_2$.

Space Group	$Cmmm$				
a (Å)	6.26(3)				
b (Å)	7.29(3)				
c (Å)	5.008(18)				
V (Å^3)	228.7(17)				
Z	2				
wR_p	0.828				
Atom	occ.	x	y	z	B_{iso} (Å^2)
Pd	1.0	0	0	0	2
C	0.5	0	0	0.4	2
N	0.5	0	0	0.4	2
C	0.25	0.2	0.2	0	2
N	0.25	0.2	0.2	0	2

Supplementary Table 5: Crystallographic details determined by powder X-ray diffraction for the crystalline approximant of $Pt(CN)_2$.

Space Group	$Cmmm$				
a (Å)	6.652(16)				
b (Å)	7.429(9)				
c (Å)	5.088(4)				
V (Å^3)	251.4(7)				
Z	2				
wR_p	3.325				
Atom	occ.	x	y	z	B_{iso} (Å^2)
Pt	1.0	0	0	0	2
C	0.5	0	0	0.4	2
N	0.5	0	0	0.4	2
C	0.25	0.2	0.2	0	2
N	0.25	0.2	0.2	0	2

Supplementary Notes

1 Neighbourhood geometries

For high-symmetry lattices, one method of constructing the neighbourhood is as follows. The Dirichlet-Voronoi[15–17] cell is identified and augmented to include those neighbouring lattice points to which the node at the centre of the cell is connected. Note that the Dirichlet-Voronoi cells provide a dense covering of space, and so the neighbourhoods also provide a dense covering. The difference is that the neighbourhood tiling is an overlapping tiling, where the overlap corresponds to the regions of augmentation. This is essentially a geometric means of enforcing "matching rules" as implemented in other aperiodic tilings of 2- and 3-space.[18] Note that, by design, the point symmetries of neighbourhood and lattice node coincide. Dirichlet-Voronoi constructions of neighbourhoods for the various high-symmetry lattices considered in our study are given in Supplementary Figures 38 and 39.

For more complex lattice geometries, it is possible that the Dirichlet-Voronoi cell cannot be used to identify the correct neighbourhood. This is the case, for example, whenever connected lattice points do not share Voronoi faces. In such cases, we anticipate that other fundamental tilings of the underlying lattice—such as affine transformations of the Voronoi decomposition—will likely provide the relevant starting point for neighbourhood identification.

2 Procrystalline system notation

In order to facilitate discussion of specific procrystalline states, we have devised a working notation which aims to identify succinctly the relevant distinguishing features of each state. Each descriptor is of the form $\mathbf{A}_n(\mathbf{B}_\mathbf{C})$, where

- **A** denotes the underlying neighbourhood lattice,
- n gives the number of linkers distinguished for each node,
- **B** clarifies the geometric arrangement of those linkers, where necessary, and
- **C** identifies the use of chiral ('X') or racemic ('R') arrangements, where necessary.

A list of corresponding terms that we have used in this document is given in Supplementary Table 2. We make no claim that this notation is anything but a working system, devised to facilitate our own book-keeping. There are obvious limitations: the nomenclature is not unique since *e.g.* $\mathbf{S_1}$ and $\mathbf{S_3}$ identify the same procrystalline state; moreover, the existence of vernacular terms (and hence abbreviations) for specific geometries is only guaranteed for high-symmetry lattices.

3 $Pd(CN)_2$ and $Pt(CN)_2$ – Background

The structures of platinum(II) cyanide and platinum(II) cyanide have long been predicted to be related to the layered structure of $Ni(CN)_2$.[19] This structure arises from the connection of square planar $[M(CN)_4]^{2-}$ units into grid-like layers; for $Ni(CN)_2$ these layers stack in the **c** crystallographic direction with no true periodicity, but such that cyanide ions of one layer are positioned directly above Ni^{2+} cations of the layer below.[20,21] Periodicity is lost due the the fact that there are twice as many CN groups as Ni atoms, such that there are two possible arrangements for each pair of layers.[20]

Intriguingly, a similar structural model is not found to account for the diffraction patterns of $Pd(CN)_2$ and $Pt(CN)_2$ due to the absence of sharp $(00l)$ peaks.[14] Rather, a very broad diffraction pattern is observed for both compounds, and for the mixed-metal cyanide $Pd_{1/2}Pt_{1/2}(CN)_2$. As noted in Ref. 14, these broadened peak profiles make structure determination from powder diffraction data alone difficult. Using a combination of powder diffraction, total scattering and spectroscopic data the authors of Ref. 14 proposed a nanocrystalline model in which the crystallite dimensions in the c direction are very small—therefore broadening the $(00l)$ reflections such that they are not seen in the diffraction pattern—but the tetragonal average symmetry of $Ni(CN)_2$ is retained.[14] The broadening term used was so severe that the corresponding structural interpretation is that all Pt/Pd cyanides should be considered completely delaminated. Analysis of the experimentally determined pair distribution functions ($T(r)$) confirms the square-planar nature of the local bonding arrangement with significant broadening in the third dimension. An off-set stacking model (retaining *p*4*mm* sheet symmetry) was used to model the PDFs, though now with finite correlations in the stacking direction.[14]

Considering the alternative model where neighbouring layers are in register, i.e. metals above each other, creates a series of metal nodes that form a nearly cubic lattice. Decorating with the square planar units in such a way that each unit is fully connected results in a network directly related to the $\mathbf{C_4T}$ procrystalline model. We find that this model better describes the experimental powder diffraction data, as described below. Such a network might be considered a defect Prussian blue structure where known guests (NH_3 or H_2O, depending on synthesis method) can be incorporated within the structure. Peak broadening therefore arises from a combination of procrystalline correlated disorder, isotropic strain and crystallite size effects.

Supplementary Methods

1 Phonon calculations

The GULP[22] input for the conventional unit cell implementation was as follows:

```
opti conv phon
cell
   2.0 2.0 10.0  90.000000  90.000000  90.000000
fractional
Nb   core 0.0 0.0 0.0
O    core 0.5 0.0 0.0
mass O 15.0
mass Nb 92.9
space
P 4/M M M
name S2Capproximant
harm
Nb O 10.0 1.0 1.3
three
Nb O O 2.0 90.0 1.2 1.2 1.9
lin3
O Nb Nb 0.1 1 1 1.3 1.3 2.4
shrink
15 15 4
dispersion 3
0.0 0.0 0.0 to 0.5 0.0 0.0
0.5 0.0 0.0 to 0.5 0.5 0.0
0.5 0.5 0.0 to 0.0 0.0 0.0
output xyz out
output phon referencephon
output eig referenceeig
```

The GULP input was altered for mean-field supercell implementation as follows:

```
opti conv phon dynamical_matrix
cell
  60.0 60.0 2  90 90 90
fractional
Nb   core 0.0 0.0 0.0
O    core 0.0166667 0.0 0.0
O    core 0.0 0.0166667 0.0
.
.
.
mass O 15.0
mass Nb 92.9
space
P 1
name S2Capproximant
harm
Nb O 10.0  1.0 1.3
three
Nb O O 2.0 90.0 1.2 1.2 1.9
lin3
O Nb Nb 0.1 1 1 1.3 1.3 2.4
kpoints
0.0 0.0 0.0
output xyz out
output phon referencephon
output eig referenceeig
```

In the supercell implementation of the mean-field case the projection gave phonon dispersion curves identical to those obtained using the conventional unit cell calculation discussed above as shown in Supplementary Figure 40. This validates the supercell implementation as a method for explicitly including disorder in phonon dispersion curve calculations.

2 $Pd(CN)_2$ and $Pt(CN)_2$ – Extraction and refinement of data

Powder diffraction data as a function of 2θ (Cu-K$_{\alpha 1}$ radiation, $\lambda = 1.540562$ Å) were extracted[23] from Figure 2 of Ref. 14. Data were interpolated, rebinned ($\Delta 2\theta = 0.1°$) and fitted using TOPAS v4.1.[24] The average structure of the $\mathbf{C_4T}$ procrystalline $M(CN)_2$ model is unstable with respect to ferroelastic distortion;[25] the ferroelastic state is best described by space group *Cmmm*, which is a maximal subgroup of the parent *P*4/*mmm* procrystal approximant. Full structural details determined using Rietveld refinement in this space group are given in Supplementary Tables 4 and 5; data and fits are presented in Supplementary Figure 42. The powder X-ray diffraction data were of sufficient quality only to refine lattice parameters and peak shape profile variables (including a Stephens (*hkl*)-dependent broadening term[26] and preferred orientation parameters), while making use of (fixed) atomic coordinates. Consequently the fits obtained are not of the same quality as Pawley refinements, but are more meaningful in the sense that a physically-sensible structural model is used to determine intensities. We note that large errors on refined lattice parameters are expected from extracted data.

References

(1) G. Baskaran, in *Current Trends in Science: Platinum Jubilee Special* (Indian Academy of Sciences, Bangalore, 2009), pp. 279–310.

(2) Cohn, H., Kenyon, R. & Propp, J. A variational principle for domino tilings. *J. Am. Math. Soc.* **14**, 297–346 (2001).

(3) Keen, D. A. & Goodwin, A. L. The crystallography of correlated disorder. *Nature* **521**, 303–309 (2015).

(4) Yang, M. *et al.* Anion order in perovskite oxynitrides. *Nat. Chem.* **3**, 47–52 (2011).

(5) Simonov, A., Weber, T. & Steurer, W. Experimental uncertainties of three-dimensional pair distribution function investigations exemplified on the diffuse scattering from a tris-*tert*-butyl-1,3,5-benzene tricarboxamide single crystal. *J. Appl. Cryst.* **47**, 2011–2018 (2014).

(6) Blunt, M. O. *et al.* Random tiling and topological defects in a two-dimensional molecular network. *Science* **322**, 1077–1081 (2008).

(7) Anderson, P. W. Resonating valence bonds: A new kind of insulator? *Mater. Res. Bull.* **8**, 153–160 (1973).

(8) Loiseau, T., Serre, C., Huguenard, C., Taulelle, F., Henry, M., Bataille, T. & Férey, G. A rationale for the large breathing of the porous aluminum terephthalate (MIL-53) upon hydration. *Chem. Eur. J.* **10**, 1373–1382 (2004).

(9) Comès, R., Lambert, M. & Guinier, A. Désordre linéaire dans les cristaux (cas du silicium, du quartz, et des pérovskites ferroélectriques). *Acta Crystallogr. A* **26**, 244–254 (1970).

(10) Whitaker, J. M. & Greaves, C. Magnetic ordering in the pyrochlore Ho_2CrSbO_7 determined from neutron diffraction, and the magnetic properties of other RE_2CrSbO_7 phases (RE=Y, Tb, Dy, Er). *J. Solid State Chem.* **215**, 171–175 (2014).

(11) Koda, A. *et al.* Staggered magnetism in LiV_2O_4 at low temperatures probed by means of the muon Knight shift. *J. Phys.: Cond. Matt.* **17**, L257 (2005).

(12) Fairbank, V. E., Thompson, A. L., Cooper, R. I. & Goodwin, A. L. Charge-ice dynamics in the negative thermal expansion material $Cd(CN)_2$. *Phys. Rev. B* **86**, 104113 (2012).

(13) Schmidt, M. *et al.* Spin singlet formation in $MgTi_2O_4$: evidence of a helical dimerization pattern. *Phys. Rev. Lett.* **92**, 056402 (2004).

(14) Hibble, S. J. *et al.* Structures of $Pd(CN)_2$ and $Pt(CN)_2$: intrinsically nanocrystalline materials? *Inorg. Chem.* **50**, 104–113 (2011).

(15) Dirichlet, G. L. Über die Reduction der positiven quadratischen Formen mit drei unbestimmten ganzen Zahlen. *J. Reine Angew. Math.* **40**, 209–227 (1850).

(16) Voronoi, G. Nouvelles applications des paramètres continus à la théorie des formes quadratiques. Deuxiéme mèmoire. Recherches sur les parallèlloédres primitifs. *J. Reine Angew. Math.* **134**, 198–287 (1908).

(17) Blatov, V. A. Voronoi-dirichlet polyhedra in crystal chemistry: theory and applications. *Cryst. Rev.* **10**, 249–318 (2004).

(18) Goodman-Strauss, C. Matching rules and substitution tilings. *Ann. Math.* **147**, 181–223 (1998).

(19) Pauling, L. *The Nature of the Chemical Bond* (Cornell University Press, Ithaca, NY, 1966).

(20) Goodwin, A. L. *et al.* Aperiodicity, structure, and dynamics in $Ni(CN)_2$. *Phys. Rev. B* **80**, 054101 (2009).

(21) Hibble, S. J., Chippindale, A. M., Pohl, A. H. & Hannon, A. C. Surprises from a simple Material—the structure and properties of nickel cyanide. *Angew. Chem. Int. Ed.* **46**, 7116–7118 (2007).
(22) Gale, J. D. GULP: A computer program for the symmetry-adapted simulation of solids. *J. Chem. Soc., Faraday Trans.* **93**, 629–637 (1997).
(23) Rohatgi, A. `http://arohatgi.info/WebPlotDigitizer` (Last checked Jul. 2015).
(24) Coelho, A. A. TOPAS-Academic, version 4.1 (Computer Software). Coelho Software, Brisbane.
(25) Hunt, S. J. *et al.* Flexibility transition and guest-driven reconstruction in a ferroelastic metal–organic framework. *Cryst. Eng. Comm.* **17**, 361–369 (2015).
(26) Stephens, P. W. Phenomenological model of anisotropic peak broadening in powder diffraction. *J. Appl. Cryst.* **32**, 281–289 (1999).
